# Pharmacodynamics (PD), Pharmacokinetics (PK) and PK-PD Modeling of NRF2 Activating Dietary Phytochemicals in Cancer Prevention and in Health

**DOI:** 10.1007/s40495-024-00388-6

**Published:** 2024-12-05

**Authors:** Ahmad Shannar, Pochung Jordan Chou, Rebecca Peter, Parv Dushyant Dave, Komal Patel, Yuxin Pan, Jiawei Xu, Md Shahid Sarwar, Ah-Ng Kong

**Affiliations:** 1 Department of Pharmaceutics, Ernest Mario School of Pharmacy, Rutgers, The State University of New Jersey, 160 Frelinghuysen Road, Piscataway, NJ 08854 USA; 2 Graduate Program in Pharmaceutical Sciences, Ernest Mario School of Pharmacy, Rutgers, The State University of New Jersey, Piscataway, NJ 08854 USA

**Keywords:** Phytochemicals, NRF2, Oxidative Stress, Inflammation, Pharmacokinetics, Pharmacodynamics

## Abstract

**Purpose of Review:**

Dietary phytochemicals, bioactive compounds derived from plants, have gained increasing attention for their potential role in cancer prevention. Among these, NRF2 (nuclear factor erythroid 2–related factor 2) activating dietary phytochemicals such as curcumin, sulforaphane, ursolic acid, and cyanidin have demonstrated significant antioxidant and anti-inflammatory properties, making them promising agents in chemoprevention. This review examines the pharmacokinetic (PK) and pharmacodynamic (PD) profiles of these dietary phytochemicals, with a focus on their NRF2-mediated effects in cancer prevention.

**Recent Findings:**

Preclinical studies have highlighted the potential of these dietary phytochemicals to modulate oxidative stress and inflammation, key drivers of carcinogenesis. We explore the complexity of their PK/PD properties, influenced by factors such as bioavailability, metabolism, and drug interactions. While most of these phytochemicals follow two compartmental PK, their anti-oxidant and anti-inflammatory effects follow the indirect response (IDR) model. Furthermore, we discuss the application of physiologically based pharmacokinetic (PBPK) modeling to simulate the behavior of these compounds in humans, providing insights for clinical translation.

**Summary:**

The integration of PK-PD analysis into the development of dietary phytochemical-based therapies offers a pathway to optimize dosing strategies, enhance therapeutic efficacy, and improve safety. This review underscores the importance of these compounds as part of cancer interception strategies, particularly in the early stages of cancer development, where they may offer a natural, less toxic alternative to conventional therapies.

**Graphical Abstract:**

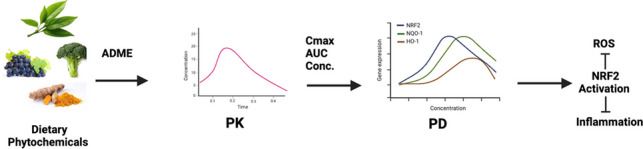

## Introduction

Cancer remains the leading cause of death worldwide, accounting for approximately 10 million deaths in 2020 (WHO). Cancer is a complex disease and cancer development takes 10–50 years involving multistep processes of initiation, promotion, progression and metastasis [1–6]. Increasing evidence suggests that ~ 80% of human cancers are linked to environmental factors (UV, radon, arsenic, asbestos, smoking, car exhaust, among others) impinging upon genetics and epigenetics [5, 7–9]. Since advanced metastasized cancers are resistant to radiation and chemotherapeutic drugs, more effective strategies in prevention of early stages of cancer and cancer interception are warranted. Dietary phytochemicals, bioactive compounds derived from plants, have emerged as promising health-promoting and disease-preventive agents due to their various health beneficial properties, chief among them antioxidant and anti-inflammatory activities [10, 11]. In particular, the use of dietary phytochemicals in cancer prevention has gained increasing attention because they offer a natural and safer alternative to traditional radiation and chemotherapy, the latter can have significant side effects [12, 13]. Phytochemicals have been shown to possess anti-inflammatory and antioxidant properties, which may contribute to their overall chemopreventive effects [14, 15]. Chronic inflammation and oxidative stress are recognized as key contributors to the development and progression of cancer, and dietary phytochemicals can modulate these processes by regulating the activity of various signaling pathways, transcription factors, and enzymes [16, 17].

NRF2 (NF-E2 p45-related factor 2) is a key transcription factor that regulates the expression of antioxidant and anti-inflammatory genes. Induction of NRF2 is deemed pivotal for cancer preventive effects of many dietary phytochemicals [18, 19]. For instance, sulforaphane, a dietary phytochemical found in cruciferous vegetables, has been shown to inhibit the expression of inflammatory markers and induce the expression of antioxidant enzymes in UVB-induced skin inflammation through the activation of NRF2 pathway [20]. Similarly, curcumin, a polyphenolic flavonoid found in turmeric, has been shown to inhibit the activation of NF-κB, a transcription factor involved in inflammation and cancer, and enhance the expression of NRF2-targeted antioxidant enzymes [21]. There are other more in-depth reviews on the complex signaling networks triggered by NRF2 activating dietary phytochemicals that would also contribute to the overall cancer preventive effects [22–24]. However, the development of phytochemical-based preventive therapies requires a more thorough understanding of their pharmacokinetic and pharmacodynamic (PK-PD) properties, which can be complex due to the chemical diversity of dietary phytochemicals, their interactions with other components of the diet, as well as the human body handling of and response to the dietary phytochemicals [25, 26].

PK-PD are essential tools for understanding the efficacy and safety of drug molecules, including dietary phytochemicals. The application of PK-PD principles to the study of dietary phytochemicals has been increasingly recognized as important driver for the development of safe and effective dietary phytochemical-based therapies [27, 28]. Phytochemicals are often present in complex mixtures, and their PK-PD can be affected by factors such as solubility, bioavailability, metabolism, and transport [29, 30]. PK-PD modeling approaches have been applied to predict the behavior of dietary phytochemicals in the body, optimize dosing regimens, and identify potential drug-drug interactions [31, 32]. Furthermore, the development of advanced analytical techniques, such as high-performance liquid chromatography and mass spectrometry (LCMS), has enabled the identification and quantification of active dietary phytochemicals and their metabolites in biological samples, providing valuable information on their PK-PD [33]. Overall, the integration of PK-PD in the study of dietary phytochemicals can greatly improve and enhance our understanding of their therapeutic potential and facilitate their development as safe and effective anticancer agents.

In this review, we will discuss the latest advances in dose–response and PK-PD of some of the most prominent dietary phytochemicals, namely curcumin (flavonoid), sulforaphane (isothiocyanate), ursolic acid (triterpenoid), and cyanidin (anthocyanidin). For the purpose of PK-PD studies and modeling, we will focus on NRF2-driven anti-oxidative and anti-inflammatory PD effects of these dietary phytochemicals in the context of cancer prevention and interception. We believe that putting together all the in vivo data on the possible anti-cancer effects of these promising dietary phytochemicals and extrapolating the dose–response relationship from animal to human will help researchers in their studies on developing phytochemicals-based cancer prevention/interception.

## Pharmacodynamics of NRF2-mediated Anti-oxidative and Anti-inflammatory by Dietary Phytochemicals

NRF2 is an emerging master regulator of cellular counteractive to oxidative stress, which can control the expression of a vast array of antioxidant response element (ARE)–dependent genes. These genes can regulate the physiological and pathophysiological outcomes of oxidative challenges [34]. The Nrf2-mediated adaptive response has been shown to mitigate toxicity and carcinogenesis during electrophilic, oxidative damage, and inflammation in animals [34, 35]. The protective characteristic of the Nrf2 pathway has been targeted for chemoprevention by the consumption of Nrf2-activating agents. This approach has been shown to decrease carcinogenesis and alter carcinogens’ metabolism in animal models and humans [36]. Therefore, NRF2 activating dietary phytochemicals, are promising agents in cancer prevention and interception.

### NRF2-ARE Anti-oxidative Pathway

Oxidative stress occurs due to an imbalance between the oxidants and antioxidant defense systems in the body which leads to many diseases, including cancer [14, 37]. Considering that cancer is a multistage process, cancer is initiated by direct attack of a carcinogen or ROS on DNA, causing genetic mutations, genome instability, and altered chromatin/DNA structure [38]. Oxidative stress can further drive DNA and histone alterations through epigenetic modifications and finally lead to the progression stage of cancer [39]. NRF2 activation, being the master regulator of phase II detoxifying and cellular antioxidant enzymes that protect against oxidative stress, would benefit host cells against cancer [40]. That beneficial effect is carried on through the Kelch‐like ECH‐associated protein 1 (Keap1)–NRF2–ARE signaling axis. This pathway regulates the transcription of several antioxidant genes (which will be further discussed below) that preserve cellular homeostasis and neutralize carcinogens before they can cause unwanted cellular and DNA damage [41].

### Role of NRF2 in Inflammation

Inflammation usually occurs when tissues are stimulated by injury or pathogens [42]. Acute inflammation, which itself is self-limiting, can clear and destroy damaged and necrotic cells and initiate tissue repair. When the injured tissue fails to heal or the healing process is dysregulated, it leads to chronic inflammation, which is one of the susceptibility factors for cellular transformation of normal cells to initiated tumor cells [43].

During chronic pathological processes, immune cells such as mast cells and monocytes are activated and recruited to the site of injury [44, 45]. At the same time, the direct infiltration of these cells and the production of a large number of inflammatory mediators would make it impossible to terminate the inflammatory response, which eventually leads to chronic inflammation and promotes tumor growth and proliferation [46, 47]. Numerous studies have shown that the transcription factor NRF2 can play a central role in regulating inflammation by affecting the expression of phase II detoxification enzymes and inflammatory mediators, thereby reducing cellular damage [48, 49].

The NRF2/heme oxygenase-1 (HO-1) axis is a therapeutic target for inflammation-related diseases. NRF2 regulates the expression of the Hmox1 gene associated with the HO-1 enzyme, which in turn affects the levels of the HO-1 enzyme and its anti-inflammatory/antioxidant metabolites such as carbon monoxide and biliverdin in vivo [50]. In one study, Nrf2/HO-1 axis was found to reduce lipid peroxidation and levels of Tnf-α and Il-6 in a rat model of intestinal tight junction dysfunction, thereby allowing inflammation to subside [51]. In mouse peritoneal macrophage-derived cells, activation of the Nrf2/Ho-1 axis interfered with intracellular lipopolysaccharide (LPS) signaling, attenuating the expression of pro-inflammatory mediators and ROS, creating a desensitized cellular phenotype and preventing excessive cellular inflammation [52].

Cytokines play an important role in a variety of inflammatory signaling responses and are broadly classified as pro- and anti-inflammatory mediators. Cell adhesion molecules (CAMs) are mainly distributed on the surface of endothelial cells and lymphocytes, and promote leukocyte recruitment, ultimately leading to tissue inflammatory damage [53]. Studies in Nrf2 knockout mice have shown that increased levels of proinflammatory cytokines (Tnf-α and Il-6) and chemokines (Mip2 and Mcp-1) can cause migration of inflammatory cells in vivo [54]. However, activated Nrf2 inhibits the activation of NF-ĸB signaling pathway and downregulates endotoxin-induced inflammatory factors transcription [55, 56]. Moreover, p38-vascular cell adhesion molecule-1 (VCAM-1) signaling has been reported to be inhibited by Nrf2, thus preventing the pro-inflammatory state of endothelial cells [57]. These findings suggest that NRF2 may be a regulator affecting the expression of cytokines and cell adhesion molecules (CAMs). Therefore, the search for new anti-inflammatory dietary phytochemicals with NRF2-dependent properties has the potential to become an important direction for research on new drugs.

### NRF2-target Genes

NRF2 regulates the expression of a wide range of genes related to antioxidant responses [58–60]. The downstream target genes are functionally divided into several categories: the first category of targets are phase I, phase II drug metabolism/detoxification enzymes, and phase III drug transporters [23]. Phase I enzymes, such as cytochromes P450 (CYP), are encoded by NRF2-regulated genes and are involved in drug metabolism pathways through redox and hydrolysis reactions [61, 62]. Phase II enzymes further modify metabolites, including glutathione S-transferases (GSTs) and UDP-glucuronosyltransferases (UGTs) [63, 64]. The endogenous or exogenous products are eventually transported out of the cell by phase III transport proteins, and all three play a synergistic role, enabling the cell to metabolize and inactivate carcinogenic/toxic xenobiotics [65].

Further studies have shown that NRF2 is also a major factor in inducing ARE-mediated activation of antioxidant and anti-inflammatory genes [66]. NRF2 can affect the expression of NAD(P)H dehydrogenase (quinone 1) (NQO1), the Superoxide dismutase (SOD) family and HO-1 encoded by the Hmox1 gene, among others [67]. Meanwhile, the presence of NRF2 reduces the expression of genes encoding pro-inflammatory factors such as interleukin 6 (IL-6) and interleukin 1β (IL-1β) [55]. In addition, many cellular processes have been demonstrated to be associated with NRF2 transcription, including metabolic reprogramming, cellular autophagy, apoptosis, protein homeostasis, and mitochondrial physiology [68]. For example, NRF2 directly induces the expression of genes that encode ferritin heavy chain (FTH) and ferritin light chain (FTL) and iron transport protein 1 (FPN1) to participate in heme and iron metabolism, regulate iron homeostasis and thus reduce the formation of harmful oxygen radicals [60, 69].

### Dietary Phytochemicals as NRF2 Activators

#### Curcumin

Curcumin, a key component of *Curcuma longa,* is a potential agent that mitigates cancer through various signaling pathways. It has been used as an adjuvant therapy in cancer patients to improve quality of life and reduce systemic inflammation [70]. Curcumin enhances NRF2 expression, stability, and nuclear translocation; thus, upregulation of HO-1 anti-oxidant gene [71]. Studies from our laboratory have shown that curcumin decreased the CpG methylation of Nrf2 and activated its expression in murine prostate cancer TRAMP-C1 cells [72]. Curcumin protects against methylmercury-induced cytotoxicity in primary rat astrocytes by increasing Nrf2 expression and cytoprotective genes NQO1 and HO-1 [73]. In sum, curcumin has been widely used as NRF2 activator and inflammatory inhibitor, which makes curcumin a promising anti-cancer dietary phytochemical [74].

#### Sulforaphane

Sulforaphane belongs to the sulfur-containing glucosinolates class of dietary phytochemicals, present in a wide variety of plant species, including cruciferous vegetables like broccoli. Due to their role in the induction of cellular detoxifying/antioxidant pathways, particularly NRF2, sulforaphane is marked as an important anti-cancer dietary phytochemical [75]. It downregulates HDAC1 and DNMT epigenetic genes and activates NRF2, while suppressing NF-κB and its target genes [76]. sulforaphane reacts with Keap1 thiol groups, releasing NRF2, stabilizing cellular NRF2, driving nuclear translocation and ARE activation and transcription of NRF2-mediated gene expression, resulting in chemoprotective effects [77]. Our lab found that topical application of sulforaphane reduced carcinogen-induced skin tumor incidence in Nrf2 WT mice, but not in Nrf2 KO mice [78]. Sulforaphane activates Nrf2 in cancer cells, sensitizing them to radiation, lowering basal ROS levels, and activating the sulforaphane-chemo-preventing mechanism [79].

#### Ursolic Acid

Ursolic acid is a pentacyclic triterpenoid derived from apple peels, guava, blueberry, cranberry, basil, rosemary, sage, thyme, and other plants [80]. It has been shown to exhibit various biological activities including anti-cancer, anti-inflammatory, anti-hypertensive, antiviral, and antioxidant [81]. Ursolic acid elicits its anticancer effect in part through antioxidative effects and epigenetic modification. Our lab has shown the potential of ursolic acid in exerting antioxidative stress and epigenetic reprogramming [82, 83]. The first study showed that knocking down Nrf2 decreased the effect of ursolic acid on protein expression of detoxifying/antioxidant genes in mouse epidermal cells [82]. The same study showed that ursolic acid can potentially reverse the hypermethylation of the Nrf2 promoter region that is prominent in cancer and neurodegenerative diseases [82] Specifically, ursolic acid diminished the protein levels of DNA methyltransferases DNMT1 and DNMT3a. The other study, done with prostate cancer xenografts, came up with the same conclusions in terms of NRF2 induction by ursolic acid through epigenetic modifications [83].

#### Anthocyanins

Anthocyanins efficacy has been studied in several in vivo mouse and rat cancer models including lung, colorectal (CRC), hepatocellular, and prostate cancers. Anthocyanins-rich natural extracts showed efficacy in suppressing angiogenesis and tumorigenesis in xenograft- and carcinogen-induced lung cancer in vivo mouse models [84–86]. These anthocyanin-driven effects are associated with inflammatory, oxidative, and metastasis signaling pathways. Furthermore, hepatocellular carcinoma rat models have been utilized to assess natural anthocyanin extracts and pure cyanidin compound protective effects and their related signaling pathways [87, 88]. Anthocyanins upregulated apoptosis and some liver detoxifying enzymes. Anthocyanins’ anti-cancer outcomes have also been observed in animal models of prostate and colorectal cancers as well [89, 90].

Tart cherry (TC) has been extensively studied for its antioxidant and anti-inflammatory properties in chronic disorders, including cancer [91, 92]. TC is a rich source of anthocyanins [93] of which cyanidin and its glycoside-derivatives, such as cyanidin-3-glucoside (C3G), cyanidin-3-glucosylrutinoside (C3GR) and cyanidin-3-rutinoside (C3R), are the main constituents [94, 95]. They have the potential to decrease intracellular ROS and DNA damage [96]. Anthocyanins are well known to modulate NRF2-regulated genes, particularly transcription of HO-1 and NQO-1 [97]. Furthermore, other cytoprotective proteins of NRF2 pathway such as SOD, thioredoxin, catalase, GST, and glutathione peroxidase are regulated by cyanidin against oxidative stress-induced disorders [98, 99]. Studies have demonstrated anthocyanin-rich beverages increase glutathione levels [100] which is long been associated with NRF2 activation [101]. Another study has reported modulation of antioxidant status and reduction of total DNA damage after 2 weeks’ consumption of anthocyanin-rich juice in healthy volunteers [102].

#### Resveratrol

Resveratrol is a polyphenol found in grapes, berries, and peanuts, widely recognized for its antioxidant and anti-inflammatory properties. It has been shown to activate the NRF2 pathway by directly interacting with the Keap1 protein, releasing NRF2 and leading to the upregulation of phase II detoxifying enzymes like HO-1 and NQO1 [103]. resveratrol role in NRF2 activation is also linked to its ability to inhibit oxidative stress, reduce inflammation, and increase the expression of antioxidant genes [104, 105]. Studies demonstrated that resveratrol prevents oxidative stress-induced cellular damage by promoting NRF2 nuclear translocation in various cancer models, including breast and prostate cancer cells [106]. Additionally, resveratrol was shown to modulate the expression of SIRT1, which is associated with NRF2 signaling, further contributing to its chemopreventive effects [107].

#### Epigallocatechin-3-gallate (EGCG)

Epigallocatechin-3-gallate (EGCG), the major catechin found in green tea, is another potent NRF2 activator with strong antioxidant and anti-inflammatory effects [108]. EGCG induces NRF2 translocation into the nucleus by disrupting the NRF2-Keap1 complex, leading to the activation of antioxidant response elements (ARE) and upregulation of detoxifying enzymes such as HO-1, NQO1, and glutathione S-transferase [109]. Furthermore, EGCG has been shown to modulate NF-κB signaling, suppressing inflammation and complementing its NRF2-mediated antioxidant actions [110]. EGCG's effects have been studied in various cancer models, where it enhanced the expression of cytoprotective genes, reduced ROS levels, and promoted apoptosis in cancer cells [111, 112].

#### Genistein

Genistein is an isoflavone predominantly found in soy products and is well known for its antioxidant and anticancer activities. Genistein activates NRF2 through the disruption of the Keap1-NRF2 complex, promoting the nuclear accumulation of NRF2 and enhancing the transcription of phase II antioxidant enzymes like HO-1 and NQO1 [113]. It also plays a role in modulating oxidative stress and reducing inflammation by inhibiting the NF-κB pathway, similar to other NRF2 activators [114]. Genistein has been shown to protect against oxidative damage in various models, including prostate and breast cancer cells, where it induced apoptosis and inhibited cell proliferation [115, 116]. Additionally, genistein's ability to demethylate the NRF2 promoter suggests epigenetic modulation as a key mechanism in its NRF2 activation [117].

## Preclinical Animal PK-PD Studies of Dietary Phytochemical

As discussed above, carcinogenesis is a long-term process in which normal cells/ tissues are transformed into cancer cells. Increased ROS and inflammation leading to changes in cancer/tumor microenvironment are considered drivers of carcinogenesis [118, 119]. Therefore, suppression of oxidative stress and inflammation using dietary phytochemicals provides us with an approach to cancer intervention. However, most studies to date are involved with in vitro cell culture models and may lack translational aspects. In the following sections, we will describe preclinical animal PK-PD studies with chemopreventive dietary phytochemicals to achieve anti-oxidative stress and anti-inflammation. Most dietary phytochemicals are ingested with food via the oral route, their systematic bioavailability (PK properties) would dictate their pharmacological effects (PD properties). Absorption, distribution, metabolism, and excretion (ADME) of dietary phytochemicals are complex processes that depend on several factors. First, the physicochemical properties of the dietary phytochemicals, such as molecular size, tendency for ionization, water and lipid solubility, and permeability. Then, the abundance of these dietary phytochemicals in the ingested diet and the form of administration (i.e., consumed as a pure compound as botanical supplement versus whole extract). Finally, the physiology and pathophysiology (including microbiota) of the body would impact the PK-PD of the phytochemical. Although all these processes and factors are interconnected with each other, we will try to break them down and discuss how PK parameters can affect the PD responses (pharmacological actions) of dietary phytochemicals. Table 1 summarizes key PK parameters of the mentioned dietary phytochemicals.

**Table 1:** Summary of pharmacokinetic characteristics of some of the dietary phytochemicals

Phytochemical	Bioavailability	Half-life (t1/2)	Major Metabolism Sites	Major Metabolites	Tissue Distribution	Formulations Available	Formulation PK Parameters	References
Curcumin	Low (< 1–3%)	3.36 h	Liver, Intestine	Dihydrocurcumin, Tetrahydrocurcumin	Liver, Kidney, Brain	Micellar, Nanoparticles	Novasol®: 185 × increase in bioavailability	[120–123]
Sulforaphane	Moderate	7.6 h	Liver	Sulforaphane-cysteine, Sulforaphane-NAC	Gastrointestinal tract, Liver	Broccoli sprout extract	Fresh sprouts: Cmax 1.9 µM, Tmax 3 h	[124–126]
Ursolic Acid	Low (~ 8%)	4.00–4.58 h	Liver, Intestine	Ursolic acid-glucuronide	Liver, Kidney	Liposomes	Intravenous: Vd 2.27 L/kg	[127–129]
Cyanidin	Very low (< 2%)	Variable (short)	Liver, Intestine	Anthocyanin metabolites	Gastrointestinal tract, Liver	Juice concentrate	High-dose juice: Cmax 19.0 ng/mL, AUC 51.1 ng*h/mL	[130–132]
Resveratrol	Low (< 1%)	1.6 h	Liver, Intestine	Glucuronide, Sulfate conjugates	Liver, Brain	Nanoparticles	Oral: Cmax < 2 µM, t1/2 1.6 h	[122, 133, 134]
EGCG	Low (~ 1.6%)	1.5–4 h	Liver, Intestine	EGCG metabolites	Liver, Colon	Green tea extract	Oral: 0.1–1.6% bioavailability	[135–137]
Genistein	Low (~ 6.8%)	46 h	Liver, Intestine	Genistein-glucuronide, sulfate	Liver, Reproductive tissues	Soy-based supplements	Oral: Cmax 0.79 µg/g	[138–140]

Several PK-PD models are available to describe the effects of anti-inflammatory agents, ranging from simple direct inhibition models to more complex mechanistic approaches like indirect response (IDR), transduction, and target-mediated drug disposition (TMDD) models. These models help in understanding how different drugs influence the inflammatory process by capturing the dynamics of drug interactions with immune cells, mediators, and disease progression over time. IDR models are widely used in PK/PD modeling to capture the time course of drug effects in inflammation by describing how a drug influences the turnover of biological mediators or cells. In these models, the drug does not act directly on the response itself but instead modifies the rates of production (k_in_) or elimination (k_out_) of these mediators [120, 133]. The general form of the IDR model equation is:

$$\frac{\mathrm{dR}}{{\mathrm{dt}}}\text{ = kin . }\left(\text{ 1 - I . }\frac{\mathrm{C}}{\text{C + IC50}} \, \right)\text{- kout} . R$$

where R is the response, k_in_ is the baseline production rate, k_out_ is the elimination rate, I represents the maximum fractional inhibition, C is the drug/phytochemical concentration, and IC_50_ is the concentration that produces 50% of the maximum inhibition/stimulation. This formulation is particularly effective in modeling drug effects that reduce the synthesis of pro-inflammatory factors or accelerate their clearance. IDR models have been used to describe processes like lymphocyte trafficking, cytokine release (e.g., TNF-α), and other immune responses in inflammatory conditions.

### Curcumin

Curcumin’s favorable health benefits urged researchers to study its PK. In a report by Boyanapalli et al. [121] lipopolysaccharide (LPS) was used to acutely trigger the inflammatory status in female Sprague–Dawley rats. curcumin (40 mg/kg) and LPS (50 μg/kg) were intravenously (i.v.) administered to suppress the inflammation and the PK-PD profile was investigated. The PK modeling indicated that the IV administration of curcumin exhibited two compartmental model. The T_1/2_ of curcumin was 3.36 h. V_C_ and V_P_ were 14.2 (L/kg) and 48.5 (L/kg), respectively. The estimated CL was 55.1 L/h/kg. The PK output of very high clearance (CL) and large volume of distribution (V_C_ + V_P_) indicated very fast metabolism and the extensive tissue distribution of curcumin. LPS induced the peak of pro-inflammatory molecules, iNos, Tnf-α, and Il-6, at approximately 3 h and curcumin successfully attenuated the inflammatory response, suggesting the powerful anti-inflammatory effects of curcumin. Jusko’s IDR model [133] with transit compartment was applied to examine the PD profile. The IDR model with the transit compartment well described the PD profiles of iNos, Tnf-α, and Il-6 in response to curcumin [121].

In another study, Wang et al. [141] investigated the antioxidant ability of two commercially marketed curcumin botanical supplements and the curcumin powder from Sigma via PK-PD modeling analysis in rats. The administration of curcumin was performed orally and intravenously. The oral dose was 250 (mg/kg) and i.v. dose was 40 (mg/kg). Once again, the plasma concentration–time profiles of the i.v. administration of the three curcumin formations were well characterized by two compartmental model. Nrf2, Ho-1, and Nqo1 were used as PD markers of antioxidant ability. Pharmacodynamically, the gene expression of Nrf2, Ho-1, and Nqo1 was evoked following the administration of the three curcumin products. The PD response of antioxidant genes was well captured by an IDR model. All formulations showed poor oral bioavailability, the absolute bioavailability of the two commercial supplements (0.9% and 0.6%) was lower than that of Sigma’s curcumin powder (3.1%) [141]. Nevertheless, oral curcumin products would elicit some PD response like Nrf2-mediated response as with the i.v. route of administration (unpublished observations). Overall, these in vivo preclinical PK-PD studies show potential health effects. Furthermore, these studies can pave the road for human clinical studies using allometric scale up. However, the very poor bioavailability of curcumin would need to be addressed before further utilization.

To overcome the above challenges and enhance the absorption of curcumin, numerous advanced pharmaceutical formulations and techniques have been investigated. These efforts can be grouped into three main groups: (i) strategies to enhance solubility, (ii) strategies to enhance permeability, and (iii) enzymatic inhibition to decrease metabolism. Strategies to enhance solubility include the use of surfactants and nanocarriers [122, 134]. Such strategies have been marketed as curcumin products because they showed high efficacy in increasing the bioavailability of curcumin. Novasol® is a marketed micellar curcumin comprises 7% curcumin and 93% Tween-80. Noasol® showed a 185-fold increase in bioavailability when compared with non-formulated curcumin in 23 healthy human volunteers [142]. Cureit® is another marketed formulation of curcumin that is based on the recreation of the natural turmeric matrix employing polar—nonpolar sandwich technology [123, 143]. Furthermore, strategies such as co-administration with piperine, a compound found in black pepper, have proven successful in enhancing curcumin's bioavailability [135]. In one study, the combination of curcumin and piperine resulted in a remarkable 2000% increase in curcumin blood levels, attributed to piperine's inhibitory effect of glucuronidation in the intestine [144].

On the other hand, curcumin distribution depends highly on its metabolism. curcumin and its metabolites, dihydrocurcumin (DHC) and tetrahydrocurcumin (THC), can be detected in plasma. However, curcumin and THC were detected in liver while curcumin and DHC were detected in kidney. Only curcumin was detected in brain, because of its high lipophilicity that enables it to cross blood brain barrier [145]. Thus, curcumin can reach brain tissue in biologically effective concentrations potentially promoting neurological health benefits. For example, upon 100 mg/kg curcumin administration via intraperitoneal injection, curcumin concentration in the brain reached between 4–5 µg/g tissue in 20–40 min time period [146]. Compared to the intraperitoneal route, mice fed with curcumin 2.5–10 mg/day for 4 months showed lower concentration in the brain (0.5 µg/g tissue) [124].

### Sulforaphane

Sulforaphane is a natural isothiocyanate, which is identified as a potent Nrf2 activator. sulforaphane triggers its antioxidative effects via the Nrf2-regulated phase II detoxifying/drug metabolizing enzymes and antioxidant enzymes [147–149]. In fact, sulforaphane is the most cited natural product activator of Nrf2, according to a recent bibliometric review [150]. However, Yagishita et al. reported, in a review of published literature of in vivo studies done on sulforaphane, that the dose selected for sulforaphane spanned more than 4-log and 3-log range for oral and intraperitoneal routes, respectively [125]. Therefore, more rigorous efforts should be considered to develop and validate biomarkers of sulforaphane PD action in humans.

In a study reported by Wang et al. the in vivo PK-PD profile and the antioxidant effect of sulforaphane were evaluated [151]. The i.v. administration of sulforaphane (25 mg/kg) in rats pharmacokinetically showed a two compartmental model. The T_1/2_ was 7.6 h, V_C_ was 1.235 (L), and CL was 0.848 (L/h). In PK-PD modeling, the IDR model was utilized to fit the response of Nrf2-mediated genes (Nrf2, Ho-1, Nqo1, Gstt1, Gpx1) in leukocytes. Pharmacodynamically, sulforaphane was able to activate the mRNA expression of Nrf2, Ho-1, Nqo1, Gstt1, and Gpx1 and peaked at 2 h and was well captured by the IDR PD model [151]. The study offers a fundamental background of sulforaphane in PK-PD translatable to human clinical studies.

### Ursolic Acid

The triterpenoid ursolic acid is rich in fruits and herbs such as cranberry and basil. It plays a role in the prevention of chronic diseases like cancer and obesity [80, 127]. Also, ursolic acid protects cells from oxidative stress via the Nrf2 signaling pathway as we discussed earlier. Despite its promising pharmacological properties, the low oral bioavailability of ursolic acid remains a significant challenge. Based on the Biopharmaceutics Classification System (BCS), ursolic acid is classified as class IV, characterized by low solubility and permeability [128].

A study by Zhang et al. revealed that the administration of ursolic acid intravenously (20 mg/kg) and orally (100 mg/kg) led to the PK profile of two-compartmental model after the i.v. route. The volume of distribution of central (V_C_) and peripheral (V_P_) compartments were 0.553 and 2.27 (L/kg), respectively. It suggested the extensive distribution of ursolic acid. In this study, the bioavailability (F) was evaluated as 8.18%. With PK-PD modeling, the IDR model with transition compartments was created to fit the time delay and gene expression of PD markers triggered by ursolic acid. Ho1, Nqo1, and Ugt1a1 mRNA expression in leukocytes were used to evaluate the antioxidative PD effects of ursolic acid. Three antioxidant genes reached the peak expression after 3–4 h of i.v. and oral administrations. LPS-induced acute inflammation rat model was utilized to evaluate the ursolic acid treatment against inflammation. The treatment of ursolic acid attenuated the proinflammatory iNos and epigenetic Dnmt1, Dnmt3a, Hdac1, and Hdac3 genes expression induced by LPS [130]. The results of the study implicate intake of ursolic acid not only triggers the mRNA levels of antioxidant genes but also alleviates the LPS-induced inflammation and epigenetic alteration.

### Anthocyanins

Anthocyanins show a very low bioavailability, with less than 2% retrieved in plasma and urine [152]. The maximal plasma concentration of cyanidin and its glycoside, the most naturally occurring anthocyanin [131], is reported to be very low, ranging from 2.3–96 nmol/L after a single dose of 188–3570 mg of cyanidin-3-glycoside [153, 154]. Although the glycosylated form of anthocyanins can be absorbed into the systemic circulation, the biological effects of these compounds have been ascribed to their metabolites, rather than the natural form [155, 156]. Anthocyanidins, for instance, can be absorbed as glycosides from the stomach and small intestine before undergoing phase-II detoxifying and metabolism transformation into more active metabolites [157, 158]. Ferrars et al. detected seventeen different metabolites of cyanidin-3-glucoside in plasma samples in human subjects [159].

### Resveratrol

Animal PK studies of resveratrol in rats show that its oral bioavailability is notably low, typically under 1%, primarily due to its extensive first-pass metabolism. Resveratrol undergoes significant metabolism primarily into glucuronide and sulfate conjugates, which are the predominant forms circulating in the bloodstream. In a single oral dose study of 2 mg/kg resveratrol in rats, peak plasma concentration was reported to be 550 ng/ml after 10 min of dosing [160]. The body disposition of resveratrol can be described in a two-compartment model with first-order linear clearance, while both metabolites were cleared by parallel first-order and Michaelis–Menten kinetics [161]. After oral administration of resveratrol in a dose range of 2–20 mg/kg, peak plasma concentration (Cmax) ranged from 0.5 to 2 µM, with a time to reach maximum concentration (Tmax) occurring within 0.5 to 1 h. The elimination half-life (T_1/2_) is relatively short, around 1 to 2 h, indicating that resveratrol is rapidly cleared from the bloodstream [136, 161].

### EGCG

The pharmacokinetics of EGCG in animal studies indicate that its absorption and bioavailability are generally low [162]. For example, in rats, the oral bioavailability of EGCG was reported to be about 1.6% when administered as pure EGCG at a dose of 75 mg/kg, and as low as 0.1% when given as part of a decaffeinated green tea extract at a dose of 200 mg/kg due to extensive first-pass metabolism [163]. In another study, the oral dose of EGCG administered to mice was 15.8 mg/kg, with a reported bioavailability of 15.8%, which highlights the differences in systemic exposure depending on the formulation and route of administration [164]. Tissue distribution studies in rats, where a single oral dose of 500 mg/kg EGCG was used, showed the highest concentrations in the small intestine mucosa (565 µM), followed by colon mucosa (69 µM), liver (48 µM), plasma (12 µM), and brain (0.5 µM), indicating significant localization in the gastrointestinal tract and metabolically active tissues [137]. These findings suggest that EGCG undergoes substantial presystemic metabolism, which limits its systemic availability, while its distribution tends to be higher in tissues associated with metabolism and excretion.

### Genistein

Genistein is a naturally occurring isoflavone predominantly found in soy and has been shown to activate NRF2, leading to antioxidant and anti-inflammatory effects. However, the pharmacokinetics of genistein in animal studies demonstrate low oral bioavailability and rapid metabolism. In FVB mice, following both intravenous and oral administration of genistein at 20 mg/kg, more than 80% of genistein was converted to glucuronides and sulfates, with an absolute bioavailability of genistein aglycone reported at 23.4% and a very long half-life of 46 h, suggesting substantial recycling or an unknown mechanism of elimination [138]. Other studies showed that genistein had poor bioavailability as the aglycone but decent bioavailability as total genistein; for example, after oral administration of 4 mg/kg genistein in Wistar rats, the absolute bioavailability of free genistein was 6.8%, while total genistein bioavailability was over 55% [139]. Tissue distribution studies indicate that the highest concentrations of genistein were found in the gut (18.5 µg/g), followed by the liver (0.98 µg/g), plasma (0.79 µg/g), and reproductive tissues, suggesting its extensive metabolism in the gastrointestinal tract and liver [140]. The extensive metabolism to glucuronides and sulfates and the high concentrations in metabolically active tissues align with its rapid processing in the body.

## Clinical Pharmacokinetic Studies of Phytochemicals

### Curcumin

In our lab’s previous work, we selected a commercial curcumin supplement and examined its PK-PD profile in healthy subjects via oral administration [165]. Each curcumin capsule consisted of 500 mg of curcuminoids and 2.5 mg of bioperine. Each study subject was provided with 8 capsules for a total dose of 4 (g) of curcuminoids and 20 (mg) of bioperine. Plasma concentrations of curcumin and its metabolite, curcumin-O-glucuronide (COG), were quantitated by LCMS. COG was used to generate the PK parameters, since the parent curcumin was below the LCMS’s limit of detection. A one compartmental model of COG well described the observed plasma data with the following PK parameters: C_max_ of 29.44 ng/mL, T_max_ of 2.53 h, T_lag_ of 0.99 h, and k_mix_ of 0.16 h^−1^ [165]. The developed Physiologically based pharmacokinetic (PBPK) modeling and simulation using Symcyp indicated the PK model of COG captured the observed data. PK-PD modeling using IDR model was utilized to describe the response of PD markers (NRF2, HO-1, NQO1, HDACs) in leukocytes with input of curcumin COG plasma concentration versus time. mRNA level of NRF2, HO-1, and NQO1 was increased while the mRNA level of HDAC1, HDAC2, and HDAC3 was decreased. Overall, the intake of curcumin supplement activated the expression of NRF2 mediated anti-oxidant genes and inhibited epigenetic gene expression of HDACs in the leukocytes of healthy subjects potentially contributing to the overall health beneficial effects of curcumin [165].

A human clinical study was performed to assess the bioavailability of the marketed Cureit® curcumin product. The study recruited 12 healthy males and the formulation resulted in a 5.5-fold AUC increase compared to unformulated powder [166]. This study concluded that Cureit® enhances the intestinal permeability of curcumin by improving physical stability and absorbability [123].

### Sulforaphane

Despite the promising outcome of sulforaphane in in vitro cell lines and in vivo murine studies, the contents of sulforaphane substantially vary in dietary vegetables because sulforaphane undergoes conversion from glucoraphanin (GFN) by the plant enzyme, myrosinase. With diverse contents of GFN and or active/inactive myrosinase, the amount of active sulforaphane in plants being absorbed into the human body would differ. Additionally, its bioavailability depends on the form of consumption. Currently, sulforaphane-rich broccoli sprout extracts (BSE) supplements are widely used as vehicles to deliver sulforaphane which appears to have higher bioavailable sulforaphane compared to GFN supplements. Therefore, in clinical studies, BSE and sulforaphane supplements are frequently used [126, 167, 168].

Atwell et al. investigated sulforaphane’s absorption and excretion of two formulations at two different dosage regimens (single dose versus divided dosing of sulforaphane) in healthy subjects [126]. Twenty healthy subjects were recruited, and they were randomized to consume fresh broccoli sprouts or a myrosinase-treated BSE (n = 10 in each group). In the single-dose group, subjects consumed 200 µmol sulforaphane equivalents of fresh broccoli sprouts or BSEs. In the divided-dose group, subjects took 100 µmol sulforaphane equivalents from fresh broccoli sprouts or BSEs twice (intake at 0 h and 12 h). Plasma and urine samples were collected and analyzed for sulforaphane and its metabolites (sulforaphane-glutathione, sulforaphane-cysteine, and sulforaphane N-acetyl-cysteine) using LCMS. sulforaphane and its metabolites showed similar T_max_ of sprouts and BSEs which was around 3 h; however, C_max_ of sprout (1.9 µM) was 2.7-fold higher than that of BSE (0.7 µM). T_1/2_ of sprouts and BSE were 3.7 h and 1.9 h, respectively. In terms of excretion, the single-dose group exhibited a peak in urine between 3 and 6 h in the sprout group while between 0 and 6 h in BSE group. Total excretion of sulforaphane metabolites in sprouts (345.7 µmol) was higher than that of BSE (109.7 µmol). While in the divided-dose group, the peaks of both sprout and BSE were observed between 0 and 3 h after the first dose. In parrel to the single-dose group, total excretion of sprout (200.1 µmol) was higher than that of BSE (67.6 µmole). This PK study revealed that fresh broccoli sprouts produced more sulforaphane than processed BSEs. Although the divided-dose group had lower AUC (area under the curve) than the single-dose group, the sulforaphane level of the divided-dose group was higher after 24 h compared to single-dose group. Pharmacodynamically, the study included mRNA and protein expression of HO-1, an NRF2-targeted gene in the whole blood. The result did not show any significant changes of these PD markers after the consumption which may due in part to the low intake of sulforaphane, rapid metabolism in cells, or analyzing the right tissue(s), to trigger the pharmacological changes [126].

Another clinical study examines the efficacy of sulforaphane-rich BSE in men with recurrent prostate cancer [169]. Twenty patients were treated with 200 μmoles/day of sulforaphane-rich extracts for a maximum period of 20 weeks. However, sulforaphane treatment showed a lack of efficacy in terms of PSA response; only one subject achieved the endpoint (≧50% PSA declines). Nevertheless, the PK study provided referrable PK parameters of sulforaphane in plasma; C_max_ was 36.7 (ng/ mL), T_max_ was 1.47 h, T_1/2_ was 2.55 h, CL was 315 (L/h), and V_Z_ was 1196 (L) [169].

### Ursolic Acid

Ursolic acid can intercept cancers through apoptosis and cell differentiation, inhibiting invasion and metastasis, and suppressing angiogenesis [81]. Liposome technology improves the poor bioavailability of ursolic acid; thus, it may enhance therapeutic efficacy. Ursolic acid liposomes have been studied and entering clinical trials in mainland China [129]. Qian et al. evaluated the tolerability of this formulation and the recommended dose in a multiple-dose regimen (56, 74, and 98 mg/m^2^) that was applied to subjects with advanced solid tumors. UAL was marked as safe for subjects with advanced solid tumors. The PK parameters were generated at the start (Day 1) and the end (Day 14) of the i.v. administration. The PK parameters, such as T_1/2_, V_d_, CL, AUC, T_max_, and C_max_ showed no significant difference between day 1 and day 14, implying linear kinetics. The elimination half-life was 4.00- 4.58 h, suggesting UAL was quickly eliminated from blood [129]. However, no acute or surrogate PD markers was incorporated into the trial.

### Anthocyanins

Tart cherry (TC) is rich in anthocyanins which has been studied and verified to benefit human health via antioxidant effects and anti-inflammatory effects [170]. It provides protection against cardiovascular diseases, diabetes, cancers, and neurodegenerative diseases [92, 93]. Anthocyanins exert their antioxidant effects via activation of NRF2 and inhibit the production of nitric oxide (NO) and tumor necrosis factor (TNF) [171, 172].

In a study conducted by Brunetti et al. [132] PK-PD analysis coupled with a population PK (PopPK) modeling were examined in subjects with gout consuming tart cherry juice concentrate (TCJC). Cyanidin-3-glucosylrutinoside (C3GR), a major anthocyanin in TCJC, was quantitated by LCMS as a PK marker in this study. Pharmacokinetically, oral administration of TCJC fitted to a one compartment model. In the high-dose group (120 mL of TCJC), C_max_ was 19.0 ng/mL, AUC was 51.1 ng*h/mL, and the absorption rate constant (ka) was 2.4 h^−1^. With the low dose group of TCJC (60 mL of TCJC), C_max_ was estimated as 8.3 (ng/mL) and AUC was 25.3 (ng*h/mL). No significant changes between ka, V, and CL were observed between the two different groups. However, the PK parameters for C_max_ and AUC appear to be dose-dependent while ka, V, and CL were dose-independent. The PK of C3GR after oral consumption of a high TCJC dose was well described by one compartment structural PopPK model with the first-order absorption and linear elimination phase. Additionally, a PBPK model to simulate the C3GR PK profile after different doses of TCJC described the PK profile of C3GR. With PK-PD modeling, an IDR model was used to capture the responses of mRNA expression of antioxidant and inflammatory genes in leukocytes. Following the high dose of TCJC, NRF2 and HO-1 mRNA expression increased by 1.3-fold and 1.4-fold quantitated by qPCR, respectively, with the peak effects observed at approximately 1–3 h. For the inflammatory gene expression, the mRNA level of TNF and iNOS was downregulated by 0.7-fold and 0.8-fold in the high dose group, respectively. However, the effects of the low dose of TCJC were not statistically significant among the antioxidant and inflammatory genes. Hence, consuming a high dose of TCJC (120 mL) could confer antioxidant and anti-inflammatory effects [132]. Taken together, this study provides a conceptual framework for future TC clinical studies/trials.

### Resveratrol

The increasing interest in resveratrol as a dietary phytochemical highlights its potential health benefits, as evidenced by the growing body of scientific literature [173]. However, there remains debate regarding the efficacy of resveratrol from dietary sources versus supplements, and the long-term effects of its consumption are not yet fully understood [174]. Pharmacokinetic studies on resveratrol in humans exhibit significant variability in terms of the sources and doses administered, ranging from 5 to 5000 mg. These sources include moderate red wine consumption, grape juice, resveratrol capsules, and pure piceid [175–179]. A double-blind, randomized, placebo-controlled study examined the pharmacokinetics and safety of trans-resveratrol in healthy adults [178]. Four groups of 10 subjects each (five males and five females) were administered trans-resveratrol at doses of 25, 50, 100, or 150 mg, taken six times daily for a total of 13 doses. Key pharmacokinetic results showed that Cmax of trans-resveratrol was reached between 0.8 and 1.5 h post-dose. After the 13th dose, mean Cmax values were 3.89 ng/mL (25 mg), 7.39 ng/mL (50 mg), 23.1 ng/mL (100 mg), and 63.8 ng/mL (150 mg). The mean AUC values were 3.1 ng·h/mL (25 mg), 11.2 ng·h/mL (50 mg), 33.0 ng·h/mL (100 mg), and 78.9 ng·h/mL (150 mg) with a half-life of 1–3 h after a single dose, which increased to 2–5 h with repeated dosing. Trough concentrations (Cmin) were below 1 ng/mL for the 25 and 50 mg doses, 3 ng/mL for the 100 mg dose, and below 10 ng/mL for the 150 mg dose. Additionally, bioavailability was higher after morning administration.

JOTROL™ is a micellar formulation of resveratrol (10%) that is thought to increase bioavailability of resveratrol via lymphatic system absorption. In a First-in-Human clinical study, resveratrol bioavailability was evaluated in humans following single ascending doses of up to 700 mg, using the JOTROL™ formulation [180]. After a single 500 mg dose of JOTROL™, a plasma Cmax of 455 ng/mL was observed. This is significantly higher compared to the 85 ng/mL Cmax seen with a 1 g encapsulated dose [181] and the 1942 ng/mL Cmax after a 2.5 g micronized dose (after normalization by dose) [182]. In this study, both the Cmax and area under the curve (AUC) of resveratrol increased supra-proportionally with increasing doses, suggesting enhanced absorption. Approximately 40–55% of the dose was recovered in urine as resveratrol and its three major conjugates, indicating a high level of absorption, though less than 1% of the administered drug remained intact in the plasma and urine relative to its metabolites [180].

### Genistein

Genistein has poor oral bioavailability, primarily due to its low water solubility and significant first-pass metabolism when administered orally. Genistein is metabolized primarily into glucuronide and sulfate metabolites [183]. This has limited its clinical use.

A new formulation of genistein, developed as an amorphous solid dispersion via hot melt extrusion, was tested for safety and pharmacokinetics in a Phase 1 study with 34 healthy volunteers [184]. In the single ascending dose study, participants received doses of 500–3000 mg, while in the multiple single dose study, participants received 3000 mg daily for six days. Amorphous genistein formulation was well-tolerated up to 3000 mg, with most adverse events being mild to moderate gastrointestinal issues and no dose-limiting toxicities. Bioavailability significantly increased between the 2000 mg and 3000 mg doses. Although Cmax of doses was not statistically different between 1000 and 2000 mg doses (203.8 and 239.5 ng/ml, respectively), Cmax of the 3000 mg dose was significantly higher (646.3 ng/ml). RNA sequencing revealed significant gene expression changes 8–12 h after the sixth dose, with 3000 mg identified as the potential effective human dose [184].

## Physiologically-Based Pharmacokinetic (PBPK) Modeling of Phytochemicals

PBPK modeling is a powerful tool used to predict the PK parameters of compounds in various tissues and organs of the human body. It offers several advantages for studying natural dietary phytochemicals by providing a detailed understanding of their absorption, distribution, metabolism, excretion, and pharmacodynamic processes. PBPK models can predict concentration–time profiles of dietary phytochemicals in plasma and target tissues, which is critical in understanding their efficacy and safety and contributes to better dose selection. Furthermore, PBPK modeling can extrapolate data from animal studies to humans, helping to predict human pharmacokinetics without requiring extensive clinical trials.

In terms of drug discovery and development, PBPK models accelerate drug screening by helping to predict the behavior of dietary phytochemicals early in drug discovery, saving time and resources in drug development. Recently, PBPK models for dietary phytochemicals have been utilized to overcome challenges of poor bioavailability and drug-phytochemical interaction.

A PBPK modeling study by Venkatesh et al. examined drug interactions between natural medicines and oncology drugs, focusing on constituents like curcumin, bergamottin (from grapefruit juice), and hyperforin (from St. John's wort) [185]. The model predicted interactions with oncology drugs like acalabrutinib, osimertinib, and olaparib, incorporating mechanisms such as CYP3A inhibition. While grapefruit juice and curcumin had minimal effects, St. John's wort posed a moderate interaction risk. Curcumin increased acalabrutinib exposure by 1.57-fold, while PBPK modeling by Adiwidjaja et al. found that systemic exposure to imatinib and bosutinib increased only by 10% at a standard curcumin dose of 320 mg twice daily, but significant interactions were predicted at higher doses (3.2 g and 1.6 g, respectively) [186]. This modeling framework aids in assessing the safety of combining herbal medicines with cancer treatments.

The effects of intestinal metabolism and enterohepatic circulation on bioavailability and systemic disposal of resveratrol in rats and humans could be investigated by PBPK modeling. Resveratrol is a natural polyphenol plant health hormone. The PBPK model simulation illustrated the significant contribution of intestinal first-pass metabolism to the systemic elimination of resveratrol and the differential effect of enterohepatic circulation on systemic exposure to resveratrol in rats and humans. After partial modification and verification, the PBPK model can optimize the drug delivery regimen and predict the interaction between resveratrol natural products and drugs in various clinical scenarios [187].

## Conclusion

Dietary phytochemicals possess great potential in cancer prevention and interception of early stages of cancers before they become to advanced and resistant to radiation and chemotherapy. These dietary phytochemicals exhibit anti-oxidative and anti-inflammatory properties through activation of NRF2 and other signaling pathways. Flavonoids (e.g., curcumin), isothiocyanates (e.g., sulforaphane), triterpenoids (e.g., ursolic acid), and anthocyanins (e.g., cyanidin) have been shown to be potent NRF2 activators and anti-inflammatory agents. The efficacy of pure compounds or plant extracts of dietary phytochemicals has been well-established in several in vitro cell-line and in vivo animal tumor models. However, the in vivo dose selection and extrapolation to human, and dosage form would need further optimization and consideration. Nanoparticles technology has been used recently to improve the poor oral bioavailability of some dietary phytochemicals with some success. Further animal and human PK-PD studies of dietary phytochemicals would greatly enhance and expand our basic understanding of the in vivo dose–response relationship (with appropriate PD markers) for cancer prevention and interception.

## Data Availability

No datasets were generated or analysed during the current study.
